# Comparison of Zinc Levels in Liver Cirrhosis and Evaluating the Severity Using Child-Pugh Score

**DOI:** 10.7759/cureus.87277

**Published:** 2025-07-04

**Authors:** Vidyadhari Kakumanu, Prakash G Mantur

**Affiliations:** 1 Internal Medicine, Shri B.M. Patil Medical College, Hospital and Research Centre, Bharathiya Lingayat Development Educational Association (BLDE) (Deemed to be University), Vijayapura, IND

**Keywords:** alcoholic liver disease, child-pugh score, disease severity, liver cirrhosis, micronutrients, nutritional status, zinc deficiency

## Abstract

Introduction

Liver cirrhosis represents the final common pathway for chronic liver diseases, characterized by extensive fibrosis and hepatocyte dysfunction. Zinc, an essential micronutrient with critical roles in protein synthesis, enzymatic reactions, and antioxidant defense, has been implicated in liver pathophysiology. This study aimed to evaluate serum zinc levels in patients with liver cirrhosis and correlate them with disease severity as measured by the Child-Pugh classification.

Methods

This hospital-based cross-sectional study was conducted among 85 patients with liver cirrhosis attending the outpatient and inpatient departments of BLDE University’s Shri BM Patil Medical College Hospital. Clinical evaluation and biochemical investigations were performed, including serum zinc levels and Child-Pugh scoring. Zinc deficiency is defined as serum levels below 51 μg/dL. Statistical analysis included descriptive statistics, chi-square tests, and analysis of variance (ANOVA) to assess relationships between variables.

Results

The study cohort comprised predominantly middle-aged men (95.3%), with alcoholic etiology (n=76, 89.4%) being the leading cause of cirrhosis. Advanced disease was common, with 67.1% of patients categorized as Child-Pugh Class C. Zinc deficiency was observed in 97.6% (n=83) of patients, with mean zinc levels showing a significant progressive decrease from Child-Pugh Class A (50.4±4.31 μg/dL) to Class B (42.32±4.86 μg/dL) to Class C (37.02±3.68 μg/dL) (p<0.001). There was a perfect parallelism between zinc and albumin deficiency (n=83, 97.6%). The mortality rate during the study period was 15.3% (n=13).

Conclusion

This study reveals a markedly high prevalence of zinc deficiency among patients with liver cirrhosis and establishes a significant inverse correlation between serum zinc levels and disease severity. The observed progressive decline in zinc concentrations with advancing Child-Pugh scores indicates that zinc deficiency may function both as a clinical marker of disease progression and as a contributing factor to hepatic deterioration. These findings highlight the potential value of incorporating routine zinc assessment in the evaluation of cirrhotic patients. Moreover, zinc supplementation may be considered as an adjunctive therapeutic strategy, particularly in those with advanced-stage disease.

## Introduction

Liver cirrhosis is the advanced stage of chronic liver disease, characterized by severe fibrosis and disruption of normal hepatic architecture caused by the creation of regenerating nodules. This irreversible condition represents a major global health burden worldwide, with fatality rates steadily increasing in recent decades [1]. While numerous factors influence the onset and progression of cirrhosis, multiple other studies have emphasized the role of micronutrient regulation, particularly zinc metabolism, in maintaining liver function and modulating disease progression [2]. Zinc, an essential trace element, plays a vital role in over 300 enzymatic processes and is critical for protein synthesis, immune regulation, wound repair, and antioxidant defense. The liver is a key organ in zinc metabolism, and zinc status profoundly impacts hepatic function [3]. In cirrhotic patients, zinc deficiency is alarmingly common, with prevalence rates reported between 52% and 88%, depending on disease severity [4].

The link between zinc deficiency and liver cirrhosis involves a complex network of pathophysiological mechanisms. Cirrhotic patients frequently exhibit impaired zinc absorption, disrupted distribution, and heightened urinary zinc excretion. These disturbances stem from multiple factors, including portal hypertension, elevated inflammatory cytokines, and diminished protein synthesis [5]. The subsequent decline in zinc levels may further aggravate liver damage by intensifying oxidative stress, weakening immune function, and reducing hepatic regenerative capacity. The assessment of disease severity in liver cirrhosis remains crucial for proper patient management and prognostication. The Child-Pugh score, developed in 1964 and revised in 1973, continues to serve as one of the most widely used tools for evaluating the severity of liver dysfunction [6]. This scoring system includes five clinical parameters: serum albumin, bilirubin, prothrombin time, ascites, and hepatic encephalopathy. Patients are classified into three classes (A, B, and C) with increasing severity, which helps guide clinical decision-making and predict survival outcomes.

Studies have suggested a potential correlation between serum zinc levels and the severity of liver cirrhosis as assessed based on the Child-Pugh score [5,7]. Several studies have demonstrated that zinc deficiency becomes more pronounced as liver disease progresses, with the lowest zinc levels observed in Child-Pugh Class C patients [7]. This relationship raises essential questions about the potential role of zinc status as both a prognostic indicator and a therapeutic target in cirrhotic patients. The clinical implications of zinc deficiency in cirrhosis extend beyond direct hepatic effects. Low zinc levels have been associated with various complications commonly observed in cirrhotic patients, including hepatic encephalopathy, immune dysfunction, and impaired wound healing [8]. Moreover, some studies suggest that zinc supplementation may improve clinical outcomes in selected patients, although the evidence remains heterogeneous and sometimes contradictory [9].

Despite the growing recognition of zinc’s importance in liver cirrhosis, several knowledge gaps persist. The exact mechanisms linking zinc deficiency to disease progression remain incompletely understood. Furthermore, the optimal methods for assessing zinc status in cirrhotic patients and the potential role of zinc parameters in disease monitoring require further investigation. Questions also remain regarding the most effective strategies for zinc supplementation, including optimal dosing, timing, and patient selection criteria [10]. The current study aimed to assess and compare zinc levels with the severity of the Child-Pugh Score.

## Materials and methods

This cross-sectional study was conducted among inpatients and outpatients of the Department of General Medicine, Shri B M Patil Medical College and Research Centre, Vijayapura, Karnataka, India, from May 1, 2023 to December 25, 2024. All patients diagnosed with liver cirrhosis based on clinical examination and abdominal ultrasonography of any etiology were included. Patients who received zinc supplements and had a history or evidence of inflammatory bowel disease were excluded. Patients were recruited based on predetermined inclusion and exclusion criteria. A comprehensive medical history was recorded for each participant, followed by a thorough clinical examination. The diagnosis of liver cirrhosis was confirmed by ultrasonography. Detailed patient information was gathered using a standardized proforma, including demographic data, clinical presentations, and risk factors for liver disease. All data were recorded using a standardized case record form. To protect privacy, all collected data were anonymized before statistical analysis.

Blood samples were collected between 7:00 AM and 9:00 AM to minimize the effect of diurnal variation in serum zinc levels. Samples for zinc analysis were collected in trace element-free vacutainers, incubated at room temperature for 30 minutes, and centrifuged at 3000 rpm for 10 minutes. The separated serum was analyzed using photometry to determine zinc levels. Specific investigations included liver function tests, and blood albumin levels were measured using an automated biochemistry analyzer (Roche Cobas c311, Roche Diagnostics, Mannheim, Germany) with Roche reagent kits per the manufacturer’s protocol. Prothrombin time and the international normalized ratio (INR) were assessed using a semi-automated coagulometer. Viral markers, including hepatitis B surface antigen (HBsAg) and hepatitis C virus (HCV), were assessed using rapid immunochromatographic test kits (Trueline™, Diagnostics Enterprises, Vijayapura, Karnataka, India), with confirmatory enzyme-linked immunosorbent assay (ELISA) performed using ErbaLisa™ kits (Transasia Bio-Medicals Ltd., Mumbai, India) as needed. The zinc levels were estimated using a dedicated zinc kit using a colorimetric method (Corals, Clinical Systems, Goa, India) on a spectrophotometer. Zinc in an alkaline medium reacts with Nitro-PAPS to form a purple-colored complex; the intensity of color was directly related to the concentration of zinc present in the specimen, measured at 570 nm. The zinc deficiency was defined as <51 μg/dL by established clinical reference ranges for adults [11].

Disease severity assessment

The criteria used to categorize patients based on the Child-Pugh score are as follows: Encephalopathy is scored as None=1 point, Grades 1 and 2=2 points, and Grades 3 and 4=3 points; Ascites is scored as None=1 point, Slight=2 points, and Moderate=3 points. Bilirubin levels are scored as under 2 mg/mL=1 point, 2-3 mg/mL=2 points, and over 3 mg/mL=3 points; Albumin levels are scored as greater than 3.5 mg/mL=1 point, 2.8-3.5 mg/mL=2 points, and less than 2.8 mg/mL=3 points; and INR is scored as under 1.7=1 point, 1.7-2.2=2 points, and above 2.2=3 points [6].

Based on the total score, the severity of cirrhosis is classified as follows: Child-Pugh A: 5 to 6 points (indicating good hepatic function); Child-Pugh B: 7 to 9 points (indicating moderately impaired hepatic function); and Child-Pugh C: 10 to 15 points (indicating advanced hepatic dysfunction).

Statistical analysis

Data were collected in pro forma and analyzed using SPSS v23.0 (IBM Corp, Armonk, NY), operating on Windows 10. The data were analyzed using the chi-square test for categorical data and the t-test for continuous data. A p-value of <0.05 was considered statistically significant for all statistical purposes.

## Results

Table 1 provides an overview of the demographic and clinical characteristics of the 85 participants in the study. Of the total, men constituted a majority (81 patients, 95.3%), while females represented only 4.7% of the patients (four patients). This study’s primary cause of liver cirrhosis accounted for 89.4% of the participants (76 patients). Viral causes were much less common, with Hepatitis B responsible for 5.9% (five patients) and Hepatitis C for 4.7% (four patients). In the majority of patients, 97.6% (83 patients) had zinc deficiency (<51 μg/dL), while only 2.4% (two patients) had normal zinc levels (52-286 μg/dL). The in-hospital mortality rate was 15.3% (13 patients), while 84.7% (72 patients) survived. Most patients (67.1%) were classified as Child-Pugh Class C, followed by 28.2% in Class B, and only 4.7% in Class A.

**Table 1:** Distribution of the patients according to demographic details, severity scores, blood parameters and outcome.

Variables		Frequency	Percentage
Age (in years)	<30	6	7.1
	31-40	19	22.4
	41-50	29	34.1
	51-60	22	25.9
	>60	09	10.6
Gender	Female	4	4.7
	Male	81	95.3
Etiology	Alcohol	76	89.4
	Hepatitis B	05	5.9
	Hepatitis C	04	4.7
Alcohol consumption	<60 g/day	11	14.5
	60+ g/day	65	85.5
Child-Pugh Score	Class A	4	4.7
	Class B	24	28.2
	Class C	57	67.1
Ascites	Mild	5	5.9
	Moderate	27	31.8
	Severe	53	62.4
Zinc (μg/dL)	Zinc deficiency (<51)	83	97.6
	Normal (52-286)	2	2.4
Albumin (g/dL)	Reduced (<3.3)	83	97.6
	Normal (3.4-5.4)	2	2.4
Outcome	Alive	72	84.7
	Death	13	15.3

Table 2 shows a significant relationship (p=0.005) between zinc deficiency and disease severity. Zinc deficiency was present in three out of four patients (75%) in Class A, 23 out of 24 patients (95.8%) in Class B, and 57 out of 57 patients (100%) in Class C. Mean zinc levels decreased from Class A (50.4±4.31 μg/dL) to Class B (42.32±4.86 μg/dL) to Class C (37.02±3.68 μg/dL), indicating that zinc levels drop as liver disease worsens.

**Table 2:** Distribution of patients according to zinc Levels with Child-Pugh scores

Zinc (μg/dL)	Class A	Class B	Class C
Zinc deficiency (<51)	3 (75%)	23 (95.8%)	57 (100%)
Normal (52-286)	1 (25%)	1 (4.2%)	0
Total	4 (100%)	24 (100%)	57 (100%)
Mean+SD	50.4 + 4.31	42.32 + 4.86	37.02 + 3.68
P value	0.005*		
*P-value of <0.05 was considered statistically significant.			

**Figure 1:**
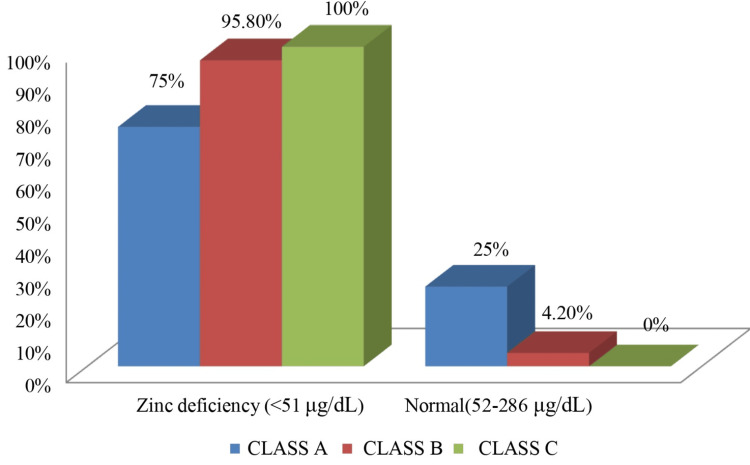
Distribution of zinc levels in liver cirrhosis patients across Child-Pugh classes Image credit: Dr. Vidyadhari Kakumanu and Dr. Prakash G. Mantur.

Figure 1 illustrates the distribution of zinc status in liver cirrhosis patients across Child-Pugh classes. Zinc deficiency (<51 μg/dL) was observed in 3/4 (75%) Class A, 23/24 (95.8%) Class B, and 57/57 (100%) Class C patients. Normal zinc levels (52-286 μg/dL) were found only in 1/4 (25%) Class A and 1/24 (4.2%) Class B patients. None of the Class C patients had normal zinc levels. This figure visually demonstrates the inverse correlation between serum zinc levels and the severity of liver dysfunction.

Table 3 demonstrates that age distribution showed a significant relationship with zinc levels (p=0.029), with all patients younger than 60 years showing zinc deficiency, while two patients older than 60 years had normal levels. Gender distribution showed no significant relationship with zinc levels (p=0.75). Etiology showed no significant relationship with zinc levels (p=0.21), as zinc deficiency was present across all etiologies.

**Table 3:** Distribution of zinc Levels across age, gender and etiology

Variable		Zinc deficiency (83)	Normal (2)	P value
Age (yrs)	<30	3 (3.5%)	0	0.029
	31-40	26 (31.3%)	0	
	41-50	27 (32.5%)	0	
	51-60	20 (24.1%)	0	
	>60	07 (8.4%)	2 (100%)	
Gender	Female	4 (4.8%)	0	0.75
	Male	79 (95.2%)	2 (100%)	
Etiology	Alcohol	74 (89.2%)	2 (100%)	0.21
	Hepatitis B	5 (6%)	0	
	Hepatitis C	4 (4.8%)	0	

Table 4 presents the signs of decompensated liver disease. The most frequently observed clinical signs included moderate to severe ascites, which were present in 80 cases (94.1%), and pedal edema, noted in 77 cases (90.6%). Jaundice was observed in 67 patients (78.8%), while hepatic encephalopathy occurred in 34 patients (40%).

**Table 4:** Signs of decompensation in liver cirrhosis Frequency and percentage have been reported for all variables.

Clinical Feature	Frequency (n)	Percentage (%)
Ascites (moderate/severe)	80	94.1
Pedal edema	77	90.6
Hepatic encephalopathy	34	40
Jaundice	67	78.8

Table 5 demonstrates the correlation between serum zinc and Child-Pugh score, showing a correlation coefficient (r) of -0.576, which was statistically significant (p<0.001). The negative correlation coefficient (r=-0.576) indicates an inverse relationship between serum zinc levels and Child-Pugh score in patients with liver cirrhosis. The strength of this correlation (-0.576) is considered moderate to strong. The correlation between serum zinc and serum albumin (r=0.326, p=0.002) was statistically significant. This positive correlation suggests that serum zinc levels also tend to decrease as serum albumin levels decrease.

**Table 5:** Correlation of serum zinc with Child-Pugh score and serum albumin

Serum zinc	Correlation coefficient	P value
Child-Pugh score	-0.576	<0.001
Serum albumin	0.326	0.002

## Discussion

This study highlights the burden of advanced liver cirrhosis in a predominantly rural and semi-urban population, characterized by late-stage presentation, a striking male predominance, and a remarkably high prevalence of zinc deficiency. A significant inverse association was observed between serum zinc levels and disease severity, suggesting a potential role for zinc in cirrhosis progression and prognosis.

The age distribution revealed a predominance of middle-aged adults, with 34.1% of patients aged 41-50 years and 25.9% aged 51-60 years. These findings are consistent with previous studies, including Sajja et al., who reported a similar concentration of cirrhotic patients in the fourth and fifth decades of life [12]. This trend likely reflects the chronic natural history of liver disease, particularly alcoholic cirrhosis, which requires prolonged exposure before advancing to decompensated stages.

Alongside the age pattern, a pronounced gender disparity was noted, with men comprising 95.3% of the study cohort. This exceeds reported male-to-female ratios in other studies, including Pazhanivel et al. (4:1) [13] and Mokdad et al., who documented global ratios between 2:1 and 3:1. [14] This imbalance may be attributed to higher alcohol consumption rates among men and potential underdiagnosis in females due to sociocultural or access-related barriers.

Clinically, the majority of patients presented with advanced liver disease: 67.1% (n=57) were classified as Child-Pugh Class C, 28.2% (n=24) as Class B, and only 4.7% (n=4) as Class A. These findings are in line with those of Deep et al. [15] and Kumar et al. [16], who similarly reported high rates of decompensated cirrhosis at presentation. Patel et al. further highlighted that among 667 patients with cirrhosis or hepatocellular carcinoma, 20% had an undiagnosed chronic liver disease, and 36% experienced diagnostic delays [17]. The advanced presentation observed in our cohort may be explained by delayed healthcare access related to geographical, financial, and educational limitations.

One clinical manifestation underscoring disease severity is the high prevalence of severe ascites. This observation aligns with Ginés et al., who identified ascites as a key feature of hepatic decompensation, occurring in 5-10% of patients with compensated cirrhosis annually [18].

Of particular concern is the extremely high prevalence of zinc deficiency, observed in 97.6% of patients, far exceeding rates reported in similar studies. Sengupta et al. [19] and Grüngreiff et al. [2] respectively noted 83% and 83.9% prevalence rates among cirrhotic populations. The elevated rate in our cohort may be attributed to poor nutritional status, the predominance of alcoholic etiology, and the advanced stage of liver disease.

Importantly, the Child-Pugh classification assessed a significant inverse correlation between serum zinc levels and cirrhosis severity (p<0.001). Mean zinc levels declined from 50.4±4.31 μg/dL in Class A to 42.32±4.86 μg/dL in Class B and 37.02±3.68 μg/dL in Class C. This trend is consistent with the existing literature. Sengupta et al. found that zinc deficiency was significantly higher among Child-Pugh B or C cirrhosis cases compared to those with Child-Pugh A or among the patients with model for end stage liver disease (MELD) scores 15 or higher; also, more than 90% of these subgroups of patients were found to be zinc-deficient [19]. Katayama et al. demonstrated that zinc levels declined proportionally with worsening Child-Pugh scores, reporting a correlation coefficient of 0.469 (p<0.001) [7]. Similarly, Stamoulis et al. found a significant negative correlation between serum zinc concentrations and Child-Pugh scores (r=-0.65, p<0.001) [4].

These findings support the potential utility of zinc as a biomarker of disease severity in cirrhosis. However, further prospective studies are necessary to determine whether zinc status can serve as a reliable predictor of clinical deterioration or is simply a marker of existing liver dysfunction. Given zinc’s role in hepatic function, antioxidant activity, and immune modulation, further research is warranted to explore its prognostic value and therapeutic potential in cirrhotic patients.

Strength

This study has several strengths. Including inpatient and outpatient participants helps to comprehensively evaluate liver cirrhosis severity across different clinical settings. A standardized methodology for patient selection, biochemical analysis, and disease severity assessment ensures reliability and minimizes bias. The study employs a well-defined statistical approach with an adequate sample size, enhancing the robustness of the findings. Additionally, quality control measures in laboratory investigations and data collection improve the accuracy and validity of the results.

Limitation

This study has several limitations that warrant consideration. First, the cross-sectional design precludes the ability to determine causality between zinc deficiency and cirrhosis severity. It remains unclear whether zinc deficiency contributes to disease progression or is merely a consequence of advanced hepatic dysfunction. Longitudinal studies incorporating serial zinc measurements are needed to clarify this relationship. Additionally, multivariate analysis was not performed, which limits the ability to adjust for potential confounding factors such as alcohol consumption, a predominant etiology in our study population. Future studies should incorporate such analyses to better understand the independent contribution of zinc deficiency to disease severity.

Second, the high prevalence of alcoholic etiology (n=76, 89.4%) and marked male predominance (n=81, 95.3%) limit the generalizability of these findings to broader cirrhotic populations. Comparative analyses across diverse etiologies, such as viral hepatitis, non-alcoholic steatohepatitis (NASH), and autoimmune liver diseases, may offer additional insights into the relationship between zinc status and liver disease progression.

Furthermore, only serum zinc levels were measured in this study. While serum zinc is commonly used in clinical practice, it may not accurately reflect total body zinc stores, particularly in inflammation or hypoalbuminemia. Low albumin levels may increase urinary zinc excretion due to zinc binding to albumin, potentially confounding serum measurements. Moreover, the transient nature of serum zinc concentrations, influenced by factors such as stress, infection, or fasting status, may further limit their reliability. More comprehensive assessments, including erythrocyte zinc concentration or urinary zinc excretion, could provide a more accurate evaluation of zinc status.

Zinc levels were not assessed over time, limiting insights into dynamic changes during disease progression or treatment. Dietary zinc intake and gastrointestinal absorption were also not evaluated as key determinants of zinc status. These factors could influence zinc levels independently of liver function and should be considered in future research.

Finally, while the association between zinc deficiency and disease severity was established, the prognostic value of zinc remains to be determined. Prospective studies are needed to examine whether zinc status can serve as a predictor of clinical complications and survival outcomes. Interventional trials exploring the therapeutic role of zinc supplementation in improving hepatic function, reducing decompensation events, and enhancing overall prognosis would be particularly valuable.

## Conclusions

The findings from this study have important clinical implications. In conclusion, zinc deficiency was observed in the vast majority of patients with liver cirrhosis and showed a significant inverse correlation with disease severity. These findings support the inclusion of serum zinc estimation in the routine evaluation of cirrhotic patients, especially those with advanced disease. Zinc supplementation may offer clinical benefits and warrants further exploration through interventional studies.
